# Effects of dietary supplementation of *Enterococcus faecium* postbiotics on growth performance and intestinal health of growing male mink

**DOI:** 10.3389/fvets.2024.1409127

**Published:** 2024-07-10

**Authors:** Lin Cao, Fengxue Sun, Qifeng Ren, Ziyi Jiang, Jian Chen, Yalin Li, Lihua Wang

**Affiliations:** College of Animal Science and Technology, Qingdao Agricultural University, Qingdao, China

**Keywords:** mink, postbiotics, *Enterococcus faecium*, nutrients digestibility, intestinal morphology, immune status, intestinal microbiota

## Abstract

Recent studies have demonstrated that postbiotics possess bioactivities comparable to those of probiotics. Therefore, our experiment aimed to evaluate the effects of postbiotics derived from *Enterococcus faecium* on the growth performance and intestinal health of growing male minks. A total of 120 growing male minks were randomly assigned to 4 groups, each with 15 replicates of 2 minks. The minks in the 4 groups were fed a basal diet supplemented with 0 (control), 0.05, 0.1, and 0.15% postbiotics derived from *E. faecium* (PEF), respectively. Compared to the control, PEF improved feed/gain (F/G) during the first 4 weeks and the entire 8 weeks of the study (*p* < 0.05); in addition, 0.1% PEF improved average daily gain (ADG) during the first 4 weeks and the entire 8 weeks of the study (*p* < 0.05), while 0.15% PEF improved ADG during the first 4 weeks of the study (*p* < 0.05). Consequently, 0.1% PEF minks displayed greater body weight (BW) at weeks 4 and 8 (*p* < 0.05), and 0.15% PEF minks had greater BW at week 4 (*p* < 0.05) than minks in the control. Furthermore, compared to the control, both 0.05 and 0.1% PEF enhanced the apparent digestibility of crude protein (CP) and ether extract (EE) (*p* < 0.05) in the initial 4 weeks, while both 0.1 and 0.15% PEF enhanced the apparent digestibility of CP and DM in the final 4 weeks (*p* < 0.05). Additionally, trypsin activity was elevated in the 0.1 and 0.15% PEF groups compared to the control (*p* < 0.05). In terms of intestinal morphology, PEF increased the villus height and villus/crypt (V/C) in the jejunum (*p* < 0.05), and both 0.1 and 0.15% PEF decreased the crypt depth and increased the villus height and V/C in the duodenum (*p* < 0.05) compared to the control group. Supplementation with 0.1% PEF increased the SIgA levels but decreased the IL-2, IL-8, and TNF-α levels in the jejunum (*p* < 0.05). Compared to the control, *E. faecium* postbiotics decreased the relative abundances of *Serratia* and *Fusobacterium* (*p* < 0.05). In conclusion, the results indicate that the growth performance, digestibility, immunity, and intestine development of minks are considerably affected by *E. faecium* postbiotics. In particular, dietary supplementation with 0.1% *E. faecium* postbiotics provides greater benefits than supplementation with 0.05 and 0.15%.

## 1 Introduction

Minks have been domesticated for approximately 100 years (1). On commercial mink farms, intestinal diseases such as enteritis and diarrhea are a considerable threat to the health, growth, and survival of minks during their developing period (2). Traditionally, antibiotics have been widely used to prevent these intestinal diseases (3), which promote the growth of the animals. However, the misuse of antibiotics has resulted in the emergence of antibiotic-resistant bacteria and genes, reducing their therapeutic efficacy against diseases in both humans and animals (4). Consequently, many countries have prohibited the use of antibiotics for growth promotion in animal feed (5). With the implementation of this ban, the search for viable alternatives to antibiotics has increasingly attracted attention.

*Enterococcus faecium* is a lactic acid bacterium recognized and approved for use as a direct-fed microbial by the Ministry of Agriculture (MOA) in China (6). Some studies have demonstrated that *E. faecium* is beneficial as a feed additive for improving growth performance (7), digestibility (8), and immunity (9), while reducing diarrhea occurrence (7) and alleviating salmonella infection (9, 10). However, some strains of *E. faecium* have been identified as opportunistic pathogens with resistance to many antibiotics (11, 12). Consequently, concerns have been raised regarding the safety of *E. faecium* as a probiotic. Furthermore, ensuring the stringent storage and transportation conditions necessary for lactic acid bacteria presents a significant challenge (13). As some studies have demonstrated that the viability of bacteria is not essential for all probiotic effects, the inactivated microorganisms and their derived fractions, termed postbiotics by the International Scientific Association for Probiotics and Prebiotics (ISAPP) (14), possess bioactivities comparable to those of live probiotic bacteria (15, 16). The beneficial impact of the probiotic is partly due to the various metabolites generated by viable probiotics (17). Therefore, it is reasonable to expect that *E. faecium* postbiotics may offer more advantages than *E. faecium* probiotics due to their higher safety and stability (18).

At present, the probiotic effects of *E. faecium* on livestock and poultry production have been extensively documented (19–21). However, there is limited research on postbiotics derived from *E. faecium*, especially in mink. The present experiment was conducted to evaluate the effects of the postbiotics derived from mink-origin *E. faecium* on male minks by analyzing growth performance, nutrient apparent digestibility, digestive enzyme activity, intestinal morphology, intestinal mucosal immunity, and gut microbiota composition.

## 2 Materials and methods

### 2.1 Ethics approval

The Animal Care and Use Committee of Animal Science and Technology at Qingdao Agricultural University reviewed and approved the experimental protocol (DKY20230524-2). This study was conducted in accordance with the ARRIVE 2.0 guidelines.

### 2.2 *Enterococcus faecium* postbiotics

The strain of *E. faecium* was previously isolated from the rectal contents of mink, identified by 16S rRNA gene sequence analysis, and preserved in the China General Microbiological Culture Collection Center (No. 29262). The 16S rRNA gene sequence was deposited in the National Center for Biotechnology Information (NCBI) database under the accession number PP886227. The isolated strain of *E. faecium* was inoculated in MRS medium and cultured at 37°C for 24 h. The viable *E. faecium* in the suspension was more than 10^7^ cfu/mL by colony count. The *E. faecium* in suspension was inactivated by heat, and then the postbiotics derived from *E. faecium* were obtained. The *E. faecium* postbiotic sample was subjected to chromatographic separation using a SHIMADZU-LC30 ultra-high-performance liquid chromatography (UHPLC) system, equipped with an ACQUITY UPLC® HSS T3 (2.1 × 150 mm, 1.8 μm) (Waters, Milford, MA, United States) column. Following the UHPLC separation, the sample was analyzed using mass spectrometry with a QE Plus mass spectrometer (Thermo Scientific). Metabolomic analysis indicated that *E. faecium* postbiotics contained 28.71% organic acids and their derivatives as well as 19.01% lipids and lipid-like molecules.

### 2.3 Animals and experimental design

The experiment was carried out on a commercial mink farm in Haiyang, Yantai. A total of 120 male minks (Regal White) at 12 weeks of age with an initial body weight (IBW) of 1281.52 ± 5.98 g were randomly assigned to 4 numerically equal groups. Each group consisted of 15 replicates with 2 minks in each replicate. The minks in the 4 groups were fed a basal diet with *E. faecium* postbiotics at 0, 0.05, 0.1, and 0.15% of the diet, respectively. Our previous study (22) has demonstrated that postbiotics derived from another *Lactobacillus* species exhibit probiotic effects in minks at comparable levels of supplementation. So, similar levels of *E. faecium* postbiotics supplementation were adopted in this study. The experiment lasted 8 weeks following a 1-week adaptation period.

### 2.4 Diet and management

All minks were housed in a two-row shelter with two open sides. Two minks were kept in a cage with the dimensions of 30 × 75 × 45 cm^3^ (width × depth × height). Each cage was equipped with a wooden nest box (30 cm × 30 cm × 30 cm, L × W × H) with a metal mesh ceiling. Minks had free access to the home cage and nest box via the entrance. During the trial period, the health status of the minks was checked twice daily, and any minks with poor health or compromised welfare were promptly removed from the study. During the period of the study, minks were fed twice per day. The paste diets were formulated from sea fishes and byproducts, chicken byproducts, and egg products. The composition of the experimental diets and the nutrient levels are presented in Table 1. Each cage was equipped with one drinker, and minks had *ad libitum* access to drinking water by the drinker. The ambient temperature was maintained at 26.24°C (± 0.05), relative humidity was 65.27% (± 0.05), and the light schedule was natural light regime throughout the study.

**Table 1:** Ingredients and nutrient composition of the basal diet (air-dry basis, %).

Items	0–4 weeks	5–8 weeks
Sea fishes and byproducts	32	32
Unhatched fertilized egg	32	32
Chicken head	20	20
Extruded corn	10	10
Lard	1	2
Soybean meal	2	2
Premix^1^	3	2
Total	100	100
*Nutrient levels*		
ME (MJ/kg)^2^	15.98	17.04
Ether extract	16.65	19.85
Crude protein	31.81	31.26
Calcium	2.47	2.59
Phosphorus	1.59	1.64

^1^The premix provided the following per kg of the diets: VA 9,000 IU, VC 40 mg, VE 20 mg, VK30.5 mg, VB15 mg, VB23 mg, VB6 2.5 mg, VB121 mg, VD3 2,000 IU, nicotinic acid 20 mg, pantothenic acid 6 mg, folic acid 0.5 mg, biotin 0.5 mg, Fe 30 mg, Zn 25 mg, Mn 10 mg, Cu 5 mg, I 0.25 mg, Se 0.2 mg. ^2^The metabolizable energy is the calculated value, and the other value is the measured value. ME is calculated using the equation ME = (0.85 × CP% × 4.5 + 0.90 × EE% × 9.5 + 0.75 × NFE% × 4.0) × 4.184, NFE (%) = 100%-CP (%)-EE (%)-ash (%).

### 2.5 Samples and data collection

#### 2.5.1 Evaluation of growth performance

Animals were individually weighed at the beginning (week 0), week 4, and week 8 of the study to determine the initial (week 0), week 4, and final (week 8) body weight. The average daily gain (ADG) of minks was calculated. During the experimental period, the feed supplied and leftovers were accurately weighed and recorded over 3 days per week. The average daily feed intake (ADFI) and feed/gain (F/G) of minks were calculated for each mink individually.

#### 2.5.2 Digestive experiment

A digestive experiment using the endogenous indicator method was performed to evaluate the apparent digestibility of nutrients at weeks 3 and 7 of the experiment. Fecal samples were collected via an inclined stainless steel plate hung under the cage. A total of 24 uncontaminated fecal samples in the four groups (with six replicates in each group) were sampled to approximately 200 g over 3 days, respectively. The 3-day fecal samples were mixed and then kept at −20°C until analysis. Meanwhile, the diets of each group were sampled daily during the 3 days before feeding the minks, then pooled to obtain representative samples, and stored at −20°C until analysis. The diet and fecal samples were air-dried at 65°C to obtain the initial moisture content. All air-dried samples were ground and passed through a 40-mesh sieve. Ground diet and fecal samples were analyzed for dry matter (DM) (GB/T 6435–2014), crude ash (GB/T 6438–2007), hydrochloric acid insoluble ash (GB/T 23742–2009), crude protein (CP) (GB/T 6432–2018), and ether extract (EE) (GB/T 6433–2006).


Nutrient apparent digestibility(%)=100%−(A1/A2)×(B2/B1)

Where A1 is the content of hydrochloric acid-insoluble ash in the diet, A2 is the content of hydrochloric acid-insoluble ash in the fecal samples, B1 is the content of a certain nutrient in the diet, and B2 is the content of a certain nutrient in the fecal sample.

#### 2.5.3 Collection and detection of intestinal samples

At the end of the study (week 8), minks (*n* = 8) from each group were randomly selected and euthanized. Approximately 2–5 g of contents of the duodenum, 5 cm sections of the duodenum and jejunum, 2–5 g of the jejunum mucosal tissue, and a rectal mucosal swab were sampled per mink.

The contents of the duodenum were centrifuged at 3500 ×*g* at 4°C for 10 min. The supernatant was used to measure the activity of α-amylase, trypsin, and lipase using assay kits (Jiancheng Bioengineering Research Institute, Nanjing, China).

The duodenum and jejunum samples were rinsed with saline and then placed into 4% paraformaldehyde fixative. After rinsing with flowing water for 24 h, the samples were dehydrated through a graded series of ethanol solutions, cleared with xylene, and embedded in paraffin wax. The samples were sectioned into 5-μm-thick sections, stained with hematoxylin and eosin (H&E), mounted with coverslips, and sealed with neutral resin for subsequent histological evaluation. The villus height and crypt depth were visualized under a light microscope (Carl Zeiss, Germany), and the images captured were analyzed using the software ZEN 2011 (Blue version). The villus height was determined from the tip of the villus to the villus–crypt junction, while the crypt depth was measured from the base of the crypt to the same junction. For each sample, the average of villus heights and crypt depths was calculated from 9 measurements taken at 3 discontinuous fields (50×), with 3 measuring points per field (23). Subsequently, the villus height to crypt depth ratio (V/C) was calculated (24).

The jejunum mucosal tissue of the mink was taken, diluted with 0.9% saline (1:9 w/v), and homogenized. The homogenate was centrifuged at 3500 ×*g* at 4°C for 10 min to obtain the supernatant, which was then analyzed for immune components, including SIgA and cytokines (IL-1β, IL-8, IL-10, IL-2, IL-6, IL-12, TNF-α, and IFN-γ). These indicators were detected using the Enzyme-Linked Immunosorbent Assay (ELISA) Kit (Jiancheng Bioengineering Research Institute, Nanjing, China), and the OD values were measured at a wavelength of 450 nm using a full-spectrum microplate reader (Tecan Austria GmbH, Grodig, Austria).

The total DNA of the rectal mucosa samples of mink was extracted using the Fast DNA Spin Kit for Soil (MP, Santa Ana, CA, United States) (22). The extracted genomic DNA was detected by 1% agarose gel electrophoresis. The primers 338F (5’-ACTCCTACGGGAGGCAGCAG-3′) and 806R (5’-GGACTACHVGGGTWTCTAAT-3′) were used to amplify the V3-V4 region of the 16S rRNA gene. PCR amplification was performed on an ABI Gene Amp PCR system 9,700 thermal cycler with a program consisting of an initial denaturation at 95°C for 3 min, followed by 27 cycles of denaturation at 95°C for 30 s, annealing at 55°C for 30 s, and extension at 72°C for 30 s, concluding with a final extension at 72°C for 10 min (25). The amplicons were excised from the 2% agarose gel, purified using the AxyPrep DNA Gel Extraction Kit (Axygen, Union City, CA, United States), and tested by 2% agarose gel electrophoresis. Quantification was performed using the QuantiFluor™-ST Blue fluorescence quantification system (Promega, Madison, WI, United States). A PE 300 library was constructed based on the Illumina MiSeq platform and sequenced using the Illumina MiSeq PE 300 platform (26).

### 2.6 Statistical analysis

The data on growth performance, nutrient digestibility, digestive enzyme activity, intestinal morphology, and jejunum mucosal immune components were expressed as means ± standard error (SE) and were analyzed using one-way ANOVA with SPSS 25.0 (SPSS Institute Inc., Chicago, USA). A *p* < 0.05 means a significant difference. Duncan’s tests were used to analyze differences between groups.

The intestinal flora data were analyzed on the I-Sanger cloud platform. FLASH 1.2.11 software was used for pair-end double-ended sequence splicing. The Spearman correlation coefficient was adopted to analyze the correlation between the intestinal flora and the immunity of minks.

## 3 Results

### 3.1 Effect of PEF on growth performance

The postbiotics of *E. faecium* had significant effects on BW, ADG, and F/G of minks during the study (*p* < 0.05; Table 2). Compared to the control minks, the minks in the 0.15% PEF group were heavier (*p* < 0.05) at week 4 of the study and had greater ADG (*p* < 0.05) during the initial 4 weeks, while the minks in the 0.1% PEF group were heavier (*p* < 0.05) at weeks 4 and 8 of the study and had greater ADG (*p* < 0.05) during the initial 4 weeks and the entire 8 weeks of the study. The minks in the PEF groups had less F/G (*p* < 0.05) than the minks in the control group during the initial 4 weeks and the entire 8-week period of the study.

**Table 2:** Effect of *Enterococcus faecium* postbiotics on growth performance (*n* = 15).

Item	Groups				*p*
	Control	0.05% PEF	0.10% PEF	0.15% PEF	
*BW*					
Week 0, g	1283.21 ± 11.89	1278.57 ± 12.50	1,290 ± 13.40	1274.29 ± 10.85	0.822
Week 4, g	1742.14 ± 29.36^b^	1800.36 ± 19.62^ab^	1846.79 ± 20.47^a^	1836.79 ± 25.54^a^	0.014
Week 8, g	2181.07 ± 44.04^b^	2272.86 ± 39.15^ab^	2366.43 ± 31.03^a^	2288.00 ± 36.19^ab^	0.012
*ADG, g*					
0–4 week	16.39 ± 0.95^b^	18.64 ± 0.66^ab^	19.89 ± 0.73^a^	20.09 ± 0.94^a^	0.010
5–8 week	15.68 ± 0.98	16.88 ± 1.23	18.56 ± 0.87	16.11 ± 0.78	0.183
0–8 week	16.03 ± 0.75^b^	17.76 ± 0.80^ab^	19.22 ± 0.59^a^	18.10 ± 0.62^ab^	0.019
*ADFI, g*					
0–4 week	268.35 ± 3.28	258.64 ± 4.24	259.68 ± 3.64	263.42 ± 3.96	0.272
5–8 week	302.09 ± 6.16	281.86 ± 6.65	279.82 ± 8.14	287.23 ± 4.8	0.082
0–8 week	285.23 ± 3.95	270.25 ± 3.97	269.75 ± 5.37	275.33 ± 3.79	0.051
*F/G*					
0–4 week	17.16 ± 1.11^a^	14.11 ± 0.55^b^	13.23 ± 0.38^b^	13.46 ± 0.60^b^	0.001
5–8 week	20.34 ± 1.39	18.23 ± 1.74	15.41 ± 0.67	18.26 ± 0.79	0.054
0–8 week	18.34 ± 0.95^a^	15.59 ± 0.68^b^	14.14 ± 0.34^b^	15.38 ± 0.43^b^	<0.001

^a,b,c^Means in the same row with different superscripts differ significantly (*p* < 0.05).

### 3.2 Effect of PEF on nutrient apparent digestibility

*Enterococcus faecium* postbiotics had significant effects on the apparent digestibility of CP, EE, and DM (*p* < 0.05, Table 3). Compared to the control, 0.05% PEF significantly improved digestibility of CP (*p* < 0.05) and EE (*p* < 0.05) during the initial 4 weeks, 0.1% PEF increased digestibility of CP (*p* < 0.05) and EE (*p* < 0.05) during the first 4 weeks as well as increased digestibility of CP (*p* < 0.05) during the final 4 weeks, and 0.15% PEF increased digestibility of CP (*p* < 0.05) and DM (*p* < 0.05) during the final 4 weeks. In addition, the 0.15% PEF group had greater digestibility of DM (*p* < 0.05) than the 0.05% PEF group during the final 4 weeks.

**Table 3:** Effects of *Enterococcus faecium* postbiotics on nutrient apparent digestibility (*n* = 6).

Item	Groups				*p*
	Control	0.05% PEF	0.10% PEF	0.15% PEF	
*0–4 weeks*					
DM, %	74.22 ± 0.79	76.02 ± 0.90	75.70 ± 1.18	74.02 ± 1.12	0.416
CP, %	86.91 ± 0.59^b^	88.62 ± 0.38^a^	89.24 ± 0.39^a^	88.11 ± 0.55^ab^	0.024
EE, %	91.18 ± 1.29^b^	95.06 ± 0.33^a^	94.95 ± 0.40^a^	93.48 ± 0.88^ab^	0.014
Ash, %	29.62 ± 3.14	35.45 ± 4.17	37.32 ± 4.33	29.63 ± 3.37	0.376
*5–8 weeks*					
DM, %	74.72 ± 0.64^c^	75.34 ± 0.97^bc^	77.34 ± 0.28^ab^	77.93 ± 0.67^a^	0.012
CP, %	85.99 ± 0.19^b^	86.92 ± 0.61^ab^	88.71 ± 0.65^a^	88.43 ± 0.94^a^	0.030
EE, %	96.55 ± 0.33	97.07 ± 0.60	97.13 ± 0.40	97.36 ± 0.32	0.601
Ash, %	19.26 ± 1.98	21.16 ± 3.19	28.76 ± 2.56	27.62 ± 2.90	0.060

DM, dry matter; CP, crude protein; EE, ether extract. ^a,b,c^Means in the same row with different superscripts differ significantly (*p* < 0.05).

### 3.3 Effect of PEF on digestive enzyme activities

*Enterococcus faecium* postbiotics had significant effects on trypsin activity (*p* < 0.05, Table 4). Compared to the control, both 0.1 and 0.15% PEF significantly increased trypsin activity (*p* < 0.05).

**Table 4:** Effects of *Enterococcus faecium* postbiotics on digestive enzyme activity (*n* = 8).

Item	Groups				*p*
	Control	0.05% PEF	0.10% PEF	0.15% PEF	
Trypsin, U/mgprot	270.16 ± 34.26^b^	359.56 ± 26.34^ab^	403.36 ± 41.75^a^	449.60 ± 38.86^a^	0.014
Lipase, U/gprot	39.35 ± 6.74	38.82 ± 6.67	43.38 ± 8.55	39.00 ± 6.37	0.963
Alpha-amylase, U/mgprot	2.02 ± 0.10	1.96 ± 0.12	2.31 ± 0.17	2.10 ± 0.09	0.239

^a,b,c^Means in the same row with different superscripts differ significantly (*p* < 0.05).

### 3.4 Effect of PEF on intestinal morphology

**Figure 1:**
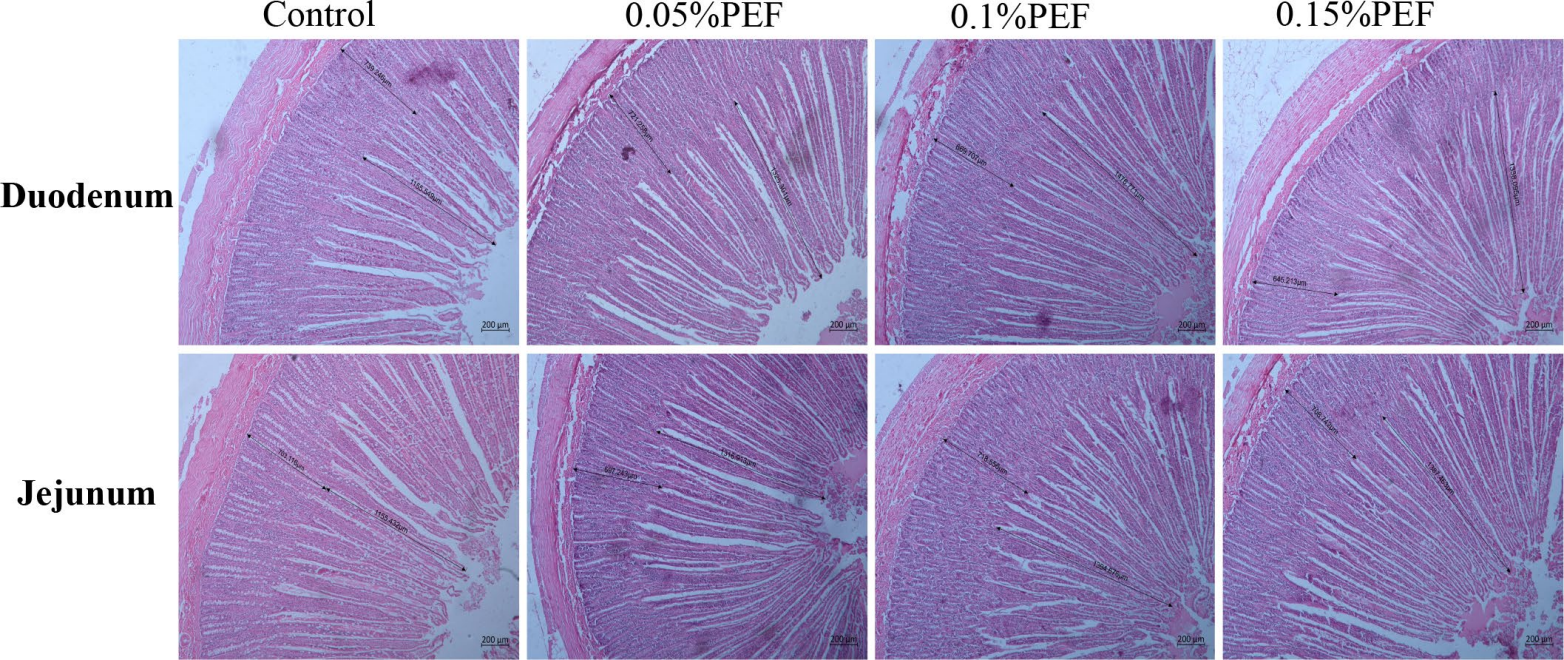
Effect of *Enterococcus faecium* postbiotics on duodenum and jejunum tissue sections of growing male minks (*n* = 8). Pictures were observed at 50 × magnification. The villus height was measured from the tip of the villus to the villus–crypt junction. The crypt depth was measured from the base of the crypt to villus–crypt junction.

*Enterococcus faecium* postbiotics had significant effects on intestinal morphology indicators (*p* < 0.05; Table 5; Figure 1). Compared to the control, 0.1% PEF increased the villus height and V/C in both the duodenum and jejunum (*p* < 0.05) and decreased the crypt depth (*p* < 0.05) in the duodenum, 0.15% PEF increased the villus height of jejunum and the V/C in both the duodenum and jejunum (*p* < 0.05) and similarly decreased the crypt depth in the duodenum (*p* < 0.05), and 0.05% PEF increased the villus height and the V/C in the jejunum (*p* < 0.05). In addition, the 0.1 and 0.15% PEF minks had less crypt depth of duodenum than the minks in the 0.05% PEF group (*p* < 0.05).

**Table 5:** Effects of *Enterococcus faecium* postbiotics on intestinal morphology (*n* = 8).

Item	Groups				*p*
	Control	0.05% PEF	0.10% PEF	0.15% PEF	
*Duodenum*					
Villus height, μm	1132.26 ± 35.87^b^	1334.21 ± 89.41^ab^	1477.76 ± 56.81^a^	1322.97 ± 115.54^ab^	0.042
Crypt depth, μm	766.25 ± 31.15^a^	757.19 ± 11.98^a^	682.68 ± 19.33^b^	653.54 ± 25.88^b^	0.004
V:C ratio	1.49 ± 0.07^b^	1.77 ± 0.13^ab^	2.18 ± 0.12^a^	2.06 ± 0.21^a^	0.008
*Jejunum*					
Villus height, μm	1124.78 ± 34.84^b^	1324.69 ± 46.75^a^	1383.37 ± 43.73^a^	1368.31 ± 43.77^a^	0.001
Crypt depth, μm	704.84 ± 35.04	690.65 ± 14.18	716.47 ± 19.90	704.09 ± 22.65	0.902
V:C ratio	1.62 ± 0.10^b^	1.92 ± 0.07^a^	1.94 ± 0.09^a^	1.95 ± 0.07^a^	0.024

^a,b,c^Means in the same row with different superscripts differ significantly (*p* < 0.05).

### 3.5 Effect of PEF on jejunum mucosal immunity indexes

*Enterococcus faecium* postbiotics had significant effects on the levels of SIgA, IL-2, IL-8, IL-10, and TNF-α in the jejunum mucosa (*p* < 0.05, Table 6). Compared to the control, 0.05% PEF decreased IL-2 (*p* < 0.05) and TNF-α (*p* < 0.05) levels, 0.1% PEF increased SIgA levels (*p* < 0.05) and decreased IL-2 (*p* < 0.05), IL-8 (*p* < 0.05), and TNF-α levels (*p* < 0.05), and 0.15% PEF decreased IL-2 levels (*p* < 0.05). Compared to the 0.1% PEF minks, the 0.05% PEF minks had less IL-10 levels (*p* < 0.05), and the 0.15% PEF minks had greater IL-8 levels (*p* < 0.05).

**Table 6:** Effects of *Enterococcus faecium* postbiotics on jejunum mucosal immunity (*n* = 6).

Item	Groups				*p*
	Control	0.05% PEF	0.10% PEF	0.15% PEF	
IL-2, pg/ml	332.49 ± 10.50^a^	278.84 ± 6.24^b^	242.57 ± 7.40^c^	270.45 ± 8.83^b^	<0.001
IL-6, pg/ml	31.38 ± 1.87	31.17 ± 2.55	29.17 ± 1.79	28.61 ± 1.99	0.717
IL-8, pg/ml	115.36 ± 1.72^a^	108.87 ± 1.03^ab^	104.02 ± 3.97^b^	112.66 ± 1.84^a^	0.019
SIgA, pg/ml	2439.30 ± 36.55^b^	2716.97 ± 109.07^ab^	2848.48 ± 122.37^a^	2420.78 ± 95.75^b^	0.015
IL-10, pg/ml	83.14 ± 2.79^ab^	70.07 ± 3.99^b^	93.51 ± 5.36^a^	82.61 ± 5.65^ab^	0.026
IL-1β, pg/ml	326.83 ± 11.94	288.61 ± 14.31	346.60 ± 26.73	297.56 ± 13.04	0.107
IFN-γ, pg/ml	1251.79 ± 120.67	1055.60 ± 69.36	1166.08 ± 111.35	1099.56 ± 156.53	0.674
TNF-α, pg/ml	774.09 ± 15.28^a^	684.02 ± 23.28^b^	693.13 ± 14.40^b^	730.32 ± 16.17^ab^	0.009

^a,b,c^Means in the same row with different superscripts differ significantly (*p* < 0.05).

### 3.6 Effect of PEF on intestinal flora

**Figure 2:**
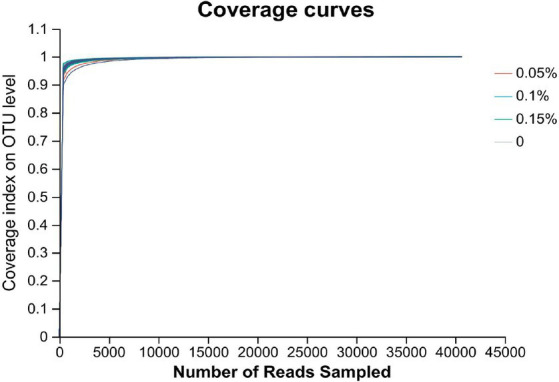
Assessment of coverage index for OUT of gut microbiota across four groups (*n* = 8).

**Figure 3:**
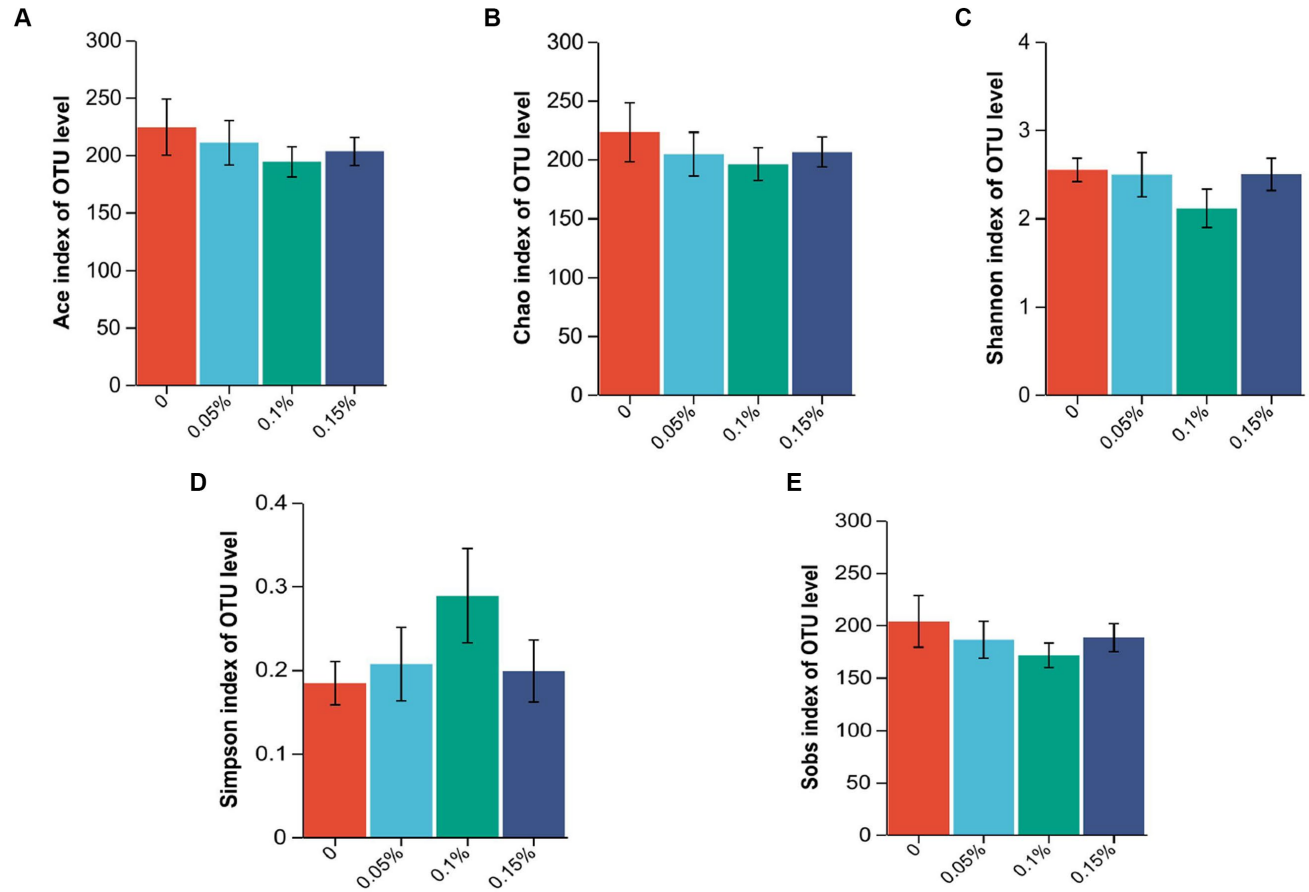
Effect of *Enterococcus faecium* postbiotics on alpha diversity indices of the growing male minks in different groups (*n* = 8). **(A)** Ace index, **(B)** Chao index, **(C)** Shannon index, **(D)** Simpson index, and **(E)** Sobs index.

The 16S rRNA sequence was assigned as an OTU with at least 97% sequence similarity. As shown in Figure 2, the end of the curve tends to be flat, indicating that the amount of sequencing data is reasonable, and all samples have sufficient sequencing depth. There were no differences among the four groups in the ACE, Chao, Shannon, Simpson, and Sobs indexes (*p* > 0.05, Figure 3).

**Figure 4:**
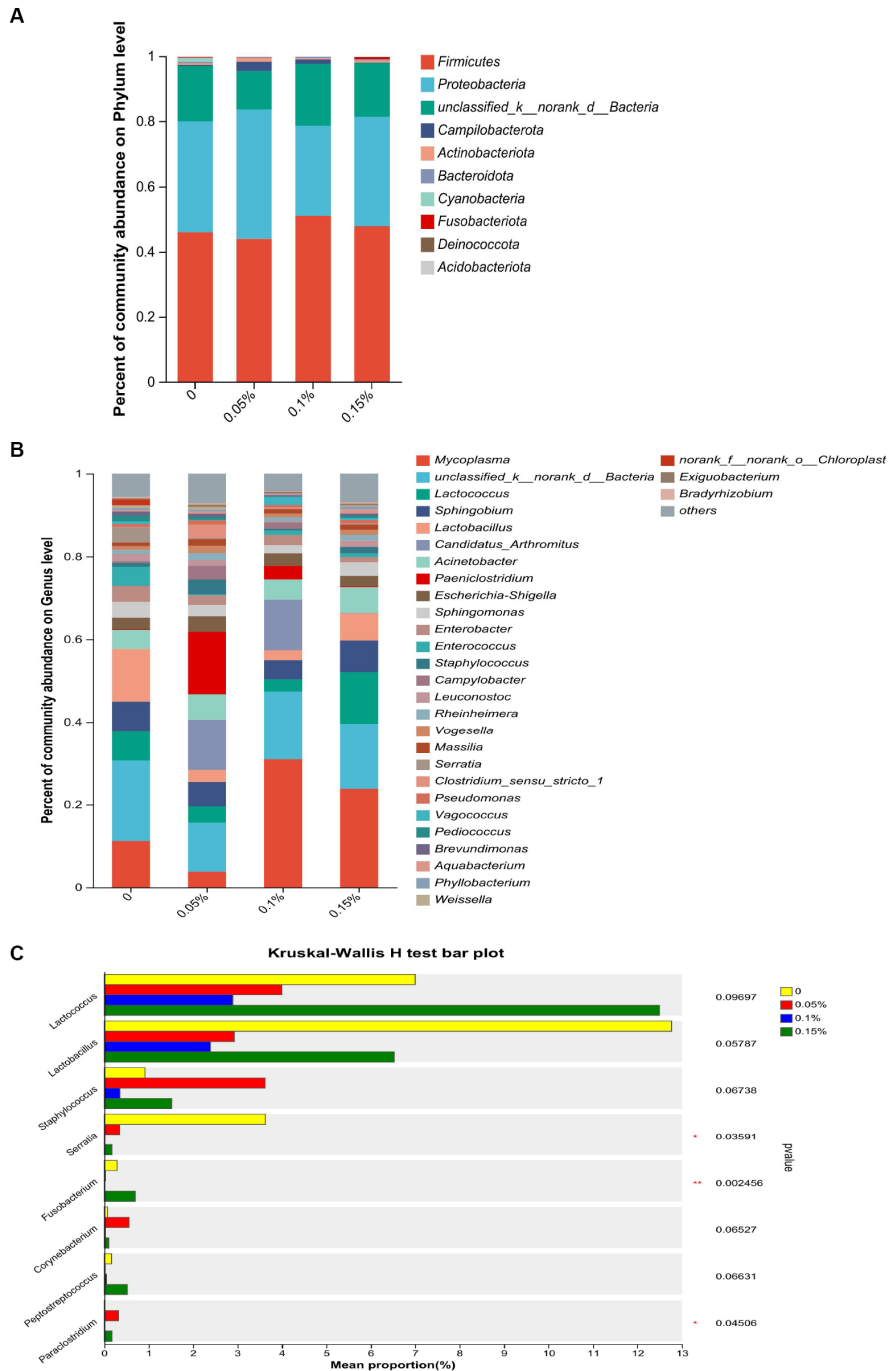
Effect of *Enterococcus faecium* postbiotics on gut microbiota composition of growing male minks. **(A)** Distribution of bacterial community structure at the phylum level (*n* = 8). **(B)** Distribution of bacterial community structure at the genus level (*n* = 8). **(C)** The significance of differences among the four groups of the same species (*represents *p* < 0.05, and ** represents *p* < 0.01). The result was statistically analyzed through non-parametric Kruskal–Wallis tests (*n* = 8).

At the phylum level, Firmicutes, Proteobacteria, and unclassified_k__norank_d__Bacteria were consistently the most abundant phyla in the four groups, which together constituted more than 96.00% of the gut microbiota (Figure 4A). At the genus level, the data obtained confirmed that *Mycoplasma*, unclassified_k__norank_d__Bacteria, *Lactococcus*, *Sphingobium*, and *Acinetobacter* were the top five dominant genera in the control group and the 0.15% PEF group (Figure 4B). *Paeniclostridium*, unclassified_k__norank_d__Bacteria, *Candidatus_Arthromitus*, *Sphingobium*, and *Acinetobacter* were the top five dominant genera in the 0.05% PEF group. *Mycoplasma*, unclassified unclassified_k__norank_d__Bacteria, *Candidatus_Arthromitus*, *Acinetobacter*, and *Sphingobium* were the top five dominant genera in the 0.1% PEF group. Further analysis of bacterial taxa among the groups indicated that the control group had a significantly higher abundance of *Serratia* than the other groups (*p* < 0.05, Figure 4C). In addition, the 0.15% PEF group displayed the highest richness of *Fusobacterium* than other groups (*p* < 0.05).

### 3.7 Correlation analysis of gut microbiota and immunity

**Figure 5:**
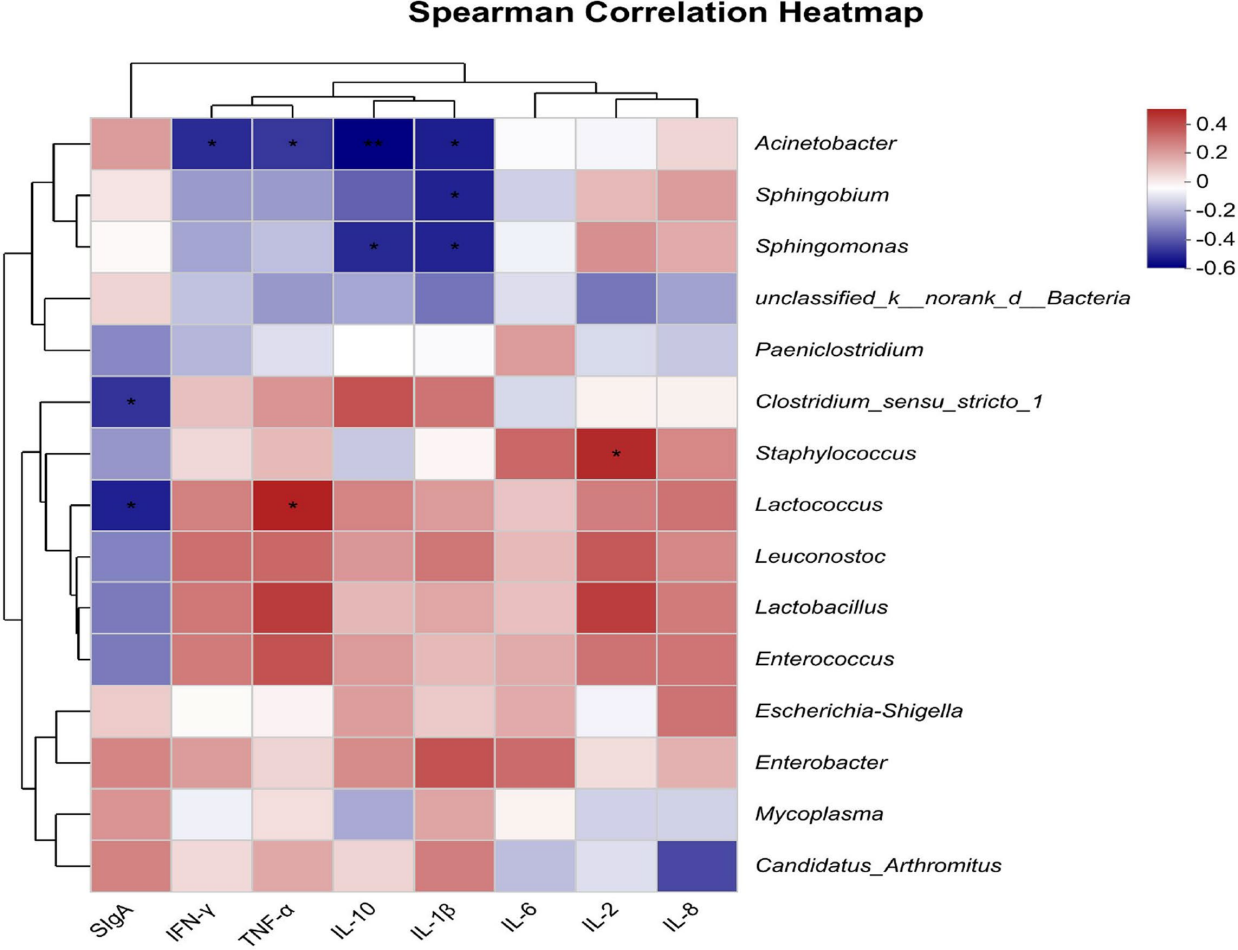
Heatmap shows the correlation between intestinal flora (genus level) and intestinal immune indicators (*n* = 8). The X-axis and Y-axis are intestinal immune indicators and species, respectively, and the correlation R-values and *p*-values are obtained through calculation. R-values are displayed in different colors in the figure. If the *p* < 0.05, they are marked with *. The legend on the right is the color range of different *R*-values; the left and upper sides present the species and immune indicator cluster trees; *represents *p* < 0.05, and **represents *p* < 0.01.

The Spearman correlation heatmap results showed that *Acinetobacter* had negative correlations with IL-1β, IL-10, TNF-α, and IFN-γ (*p* < 0.05, Figure 5). *Sphingobium* had a negative correlation with IL-1β (*p* < 0.05). *Sphingomonas* had negative correlations with IL-1β and IL-10 (*p* < 0.05). *Clostridium_sensu_stricto_1* had a negative correlation with SIgA (*p* < 0.05). *Staphylococcus* correlated positively with IL-2 (*p* < 0.05). *Lactococcus* correlated positively with TNF-α and negatively with SIgA (*p* < 0.05).

## 4 Discussion

The objective of this study was to investigate whether postbiotics derived from *E. faecium* could have potential probiotic effects on growing minks. This study demonstrated that *E. faecium* postbiotics improved the growth performance of male minks. The results were consistent with previous studies of *E. faecium* on piglets (24, 27) and broilers (28). In the current study, the increased ADG was associated with improvement of F/G, indicating that the minks in the *E. faecium* postbiotics groups were efficient in utilizing dietary nutrients for growth. This could be due to the *E. faecium* postbiotics containing many functional compounds such as short-chain fatty acids (SCFA), microbial fractions, functional proteins, secreted polysaccharides, extracellular polysaccharides (EPSs), cell lysates, and teichoic acid (29), which could improve immune function (30), inhibit pathogenic bacteria (31), promote the development of intestinal villi (32), enhance the activities of intestinal digestive enzymes (33), and improve the efficiency of nutrient utilization (24). Consequently, the growth performance of the animals was improved. The study findings confirmed that postbiotics derived from *E. faecium* at 0.1 and 0.15% were effective in enhancing both the ADG and feed efficiency of minks. In contrast, 0.05% *E. faecium* postbiotics improved feed efficiency without effect on ADG, thereby demonstrating a dose-dependent effect of the postbiotics. These results suggest that a higher dosage of postbiotic supplementation is necessary to achieve improvements in ADG. However, these improvements were only evident in the initial 4 weeks. The findings indicate that *E. faecium* postbiotics may have short-term effects on growth performance.

In the current study, *E. faecium* postbiotics increased the apparent digestibility of CP, EE, and DM. The results were consistent with previous studies on pigs, which showed that *E. faecium* improved nutrient digestibility (34, 35). Chen et al. (36) also found that *E. faecium* could improve the digestibility of DM in pigs. However, it is interesting to note that 0.05 and 0.15% *E. faecium* postbiotics only improved the apparent digestibility of CP in minks during the initial and last 4 weeks of the study, respectively. In contrast, 0.1% *E. faecium* postbiotics enhanced the apparent digestibility of CP throughout the study period. This suggests that 0.1% *E. faecium* postbiotics are the optimal dosage for improving the apparent digestibility of CP in growing minks. Omar et al. (37) suggested that the digestive enzyme activities contributed to feed utilization associated with the growth performance of animals. This may be due to the enhancement of digestive enzyme activity. Digestive enzymes break down nutrients into smaller molecules, facilitating their absorption by the animal (38). Lipases hydrolyze triglycerides into glycerol and long-chain fatty acids (39), amylase breaks down starches into monosaccharides such as glucose, and protease degrades proteins into peptides and amino acids (40). As a carnivore, the mink has a high capacity for fat digestion but a limited capacity for carbohydrate digestion due to the low activity of α-amylase (41). Furthermore, the mink has a higher protein requirement than other domestic animals (42). The increased activity of trypsin likely facilitated protein digestibility in this study. The results were consistent with the findings of previous research on fish (43) and broilers (44, 45), which showed that dietary supplementation with probiotics could increase digestive enzyme activity. It is probable that short-chain fatty acids (SCFAs) present in *E. faecium* postbiotics help maintain a healthy intestinal environment conducive to the optimal functioning of digestive enzymes. Consequently, this enhancement in nutrient digestibility improves the growth performance of the animal (46).

Furthermore, the study revealed that 0.1% *E. faecium* postbiotics increased the villus height and the V/C in both the duodenum and jejunum of minks and decreased the crypt depth in the duodenum. Several previous studies have reported that *E. faecium* probiotic or heat-killed *E. faecium* significantly increased the villus height (19, 47) and V/C (24) and decreased the crypt depth (48). The measurements of the villus height and crypt depth are indicative of gut health and function (49). To a certain extent, increases in the villus height and reductions in the crypt depth enhance digestibility and absorptivity (50). The V/C indicates the integrity of the intestinal mucosa and is associated with digestion and absorption capacity (51). These findings further elucidate that *E. faecium* postbiotics contribute to the promotion of intestinal development, thereby improving the digestibility of nutrients in minks.

In this study, 0.1% *E. faecium* postbiotics was observed to reduce IL-8, IL-2, and TNF-α levels and increase sigma levels in jejunum mucosa. However, 0.05 and 0.15% *E. faecium* postbiotics had no significant effect on the levels of IL-8 and sigma. The findings suggest that 0.1% *E. faecium* postbiotics are more effective in modulating intestinal immunity compared to the 0.05 and 0.15% supplementation. The intestinal mucosal immune system comprises lymph nodes, lamina propria, and epithelial cells, which constitute a protective barrier for maintaining intestinal integrity (52). M cells secrete SIgA through the polymeric immunoglobulin receptor in the crypts, effectively defending against the invasion of pathogens and commensal microorganisms (53, 54). As the predominant immunoglobulin in the intestine, Shiga provides immune protection to prevent the penetration of microorganisms and mucosal antigens into the mucosal barrier through immune exclusion (55). The experimental results indicated that the SIgA levels initially increased and subsequently declined with the increasing supplementation of *E. faecium* postbiotics. This suggests that *E. faecium* postbiotics may stimulate polymeric immunoglobulin receptor expression by activating pattern recognition receptors on intestinal epithelial and immune cells and increasing the concentration of SIgA in the intestinal lumen (53). While postbiotics can stimulate the immune system of the host and enhance SIgA production within an optimal dosage range, excessively high supplementation might trigger an immune suppressive or resistance, potentially resulting in a reduction of SIgA levels (56).

The intestinal epithelium can generate cytokines including IL-2, IL-8, and TNF-α, which are closely involved in triggering the inflammatory response (57). Maintaining the balance between pro-inflammatory and anti-inflammatory cytokines is essential for regulating intestinal inflammation (58). A previous study on piglets confirmed that supplementation with *E. faecium* reduced the relative expression of the IL-8 gene and the level of TNF-α in the jejunum mucosa and increased the relative expression of the IL-10 and TGF-β genes in the ileum mucosa (59). Similar results in macrophage have been reported, indicating that both live and heat-killed *E. faecium* promote IL-10 secretion and inhibit TNF-α release, respectively (60). In this study, the results indicated that *E. faecium* postbiotics could regulate immunity and inflammatory responses. A previous study confirmed that postbiotics derived from *E. faecium* SF68 could reversibly inhibit the activation of the NF-κB and JNK signaling pathway in intestinal epithelial cells and counteract the effects of bacterial and other toll-like receptors (TLRs) (61). Therefore, it is suggested that the *E. faecium* postbiotics likely regulate the immune functions of male minks by inhibiting the activation of the NF-κB and JNK signaling pathways.

The gut microbiota is associated with the metabolism, immunity, digestibility, and health of the host (62). Establishing and maintaining beneficial interactions between the host and microbiota is important to maintain host health (63). However, the results of alpha diversity in the current study did not show any effect of *E. faecium* postbiotics on the enrichment and diversity of the gut microbiota. In contrast, a previous study on piglets demonstrated that probiotic *E. faecium* increased the Sobs, Chao, ACE, and Shannon indexes and decreased the Simpson index from days 1 to 14 (20). This may be due to colonization of viable *E. faecium* on the intestinal mucosa, which contributed to the enhancement of community richness (64). In agreement with previous studies on mink, Firmicutes and Proteobacteria were identified as the most dominant phyla on the rectal mucosa in male minks, consistent with observations in the colon (65) and feces (66) of mink. In fact, Firmicutes and Proteobacteria are widely found in the gastrointestinal tract of carnivores such as otters and raccoon dogs (67). At the genus level, we observed changes in the abundance of flora. The relative abundance of *Serratia* was reduced in all PEF groups, and *Fusobacterium* showed a decrease in 0.05 and 0.1% PEF groups, respectively. *Serratia marcescens*, a member of the *Serratia* (68), is an opportunistic pathogen related to respiratory, urinary, and digestive tract infections (69). *Fusobacterium* is a gram-negative anaerobic bacterium that is typically found as part of the normal flora in the oral cavity and gastrointestinal tract (70). Several members of the *Fusobacterium* genus are opportunistic pathogens that can cause bacteremia and acute infections (71). These results illustrate that *E. faecium* postbiotics may prevent harmful bacteria from adhering to the intestinal mucosa and reduce the occurrence of inflammation.

The interaction between the intestinal microbial flora and immunity has been extensively described in many published reports (22, 72, 73). Our study specifically investigated the interaction between specific intestinal microbiota genera and gut immune indicators. We discovered a positive association between the genus *Lactococcus* and the inflammatory marker TNF-α, and an inverse relationship with SIgA. These findings suggest that *Lactococcus* may not contribute positively to mink health. The intestinal microbiome can protect the integrity of the mucosal barrier by acting on the host immune system (72), thus inhibiting the occurrence of intestinal inflammation. In addition, several previous studies suggested that alterations in microbiota may lead to immune-mediated diseases because microbial communities affect barrier surfaces as well as remote organs, including the lungs and skin (74, 75).

## 5 Conclusion

The study findings confirm that postbiotics derived from *E. faecium* exhibit probiotic effects on growing male minks. In particular, dietary supplementation with 0.1% *E. faecium* postbiotics improves growth performance (ADG and F/G during the initial 4 weeks and the entire 8 weeks of the study), the apparent digestibility of nutrients (CP, EE, and DM), and impacts immune status and intestinal morphology in the minks. Therefore, it can be concluded that supplementation with 0.1% provides greater benefits than supplementation with 0.05 and 0.15%.

## Data availability statement

The data presented in the study are deposited in the National Center for Biotechnology Information (NCBI) repository, accession number PP886227.

## Ethics statement

The animal study was approved by the Animal Care and Use Committee of Animal Science and Technology at Qingdao Agricultural University. The study was conducted in accordance with the local legislation and institutional requirements.

## Author contributions

LC: Data curation, Writing – original draft, Writing – review & editing. FS: Investigation, Writing – original draft. QR: Formal analysis, Investigation, Writing – review & editing. ZJ: Formal analysis, Writing – review & editing. JC: Conceptualization, Methodology, Writing – review & editing. YL: Conceptualization, Methodology, Writing – review & editing. LW: Funding acquisition, Supervision, Writing – review & editing.
